# Path Integral Calculation of the Hydrogen/Deuterium Kinetic Isotope Effect in Monoamine Oxidase A-Catalyzed Decomposition of Benzylamine

**DOI:** 10.3390/molecules24234359

**Published:** 2019-11-28

**Authors:** Mateusz Z. Brela, Alja Prah, Marek Boczar, Jernej Stare, Janez Mavri

**Affiliations:** 1 Faculty of Chemistry, Jagiellonian University, 30-387 Kraków, Poland; brela@chemia.uj.edu.pl (M.Z.B.); boczar@chemia.uj.edu.pl (M.B.); 2 National Institute of Chemistry, SI–1000 Ljubljana, Slovenia; alja.prah@ki.si (A.P.); jernej.stare@ki.si (J.S.); 3 Faculty of Pharmacy, University of Ljubljana, SI-1000 Ljubljana, Slovenia

**Keywords:** monoamine oxidase, kinetic isotope effect, quantum classical path

## Abstract

Monoamine oxidase A (MAO A) is a well-known enzyme responsible for the oxidative deamination of several important monoaminergic neurotransmitters. The rate-limiting step of amine decomposition is hydride anion transfer from the substrate α–CH2 group to the N5 atom of the flavin cofactor moiety. In this work, we focus on MAO A-catalyzed benzylamine decomposition in order to elucidate nuclear quantum effects through the calculation of the hydrogen/deuterium (H/D) kinetic isotope effect. The rate-limiting step of the reaction was simulated using a multiscale approach at the empirical valence bond (EVB) level. We applied path integral quantization using the quantum classical path method (QCP) for the substrate benzylamine as well as the MAO cofactor flavin adenine dinucleotide. The calculated H/D kinetic isotope effect of 6.5 ± 1.4 is in reasonable agreement with the available experimental values.

Academic Editor: Poul Erik Hansen

## 1. Introduction

Understanding the mechanisms underlying neurodegenerative and neuropsychiatric diseases, such as depression, Alzheimer’s disease, obsessive disorders, and Parkinson’s disease, is of prime importance for the development of novel drugs [1,2]. A variety of factors are involved in the development of neurological illnesses, and among these factors, enzymes play a pivotal role. In particular, the focus is on acetylcholinesterase, butyrylcholinesterase, and monoamine oxidases A and B (MAOs) [3,4].

MAOs are flavoenzymes found on the outer mitochondrial membrane of cells. The reaction they catalyze is the oxidative deamination of biogenic and dietary monoamines such as dopamine, serotonin, histamine, noradrenaline, and phenylethylamine. MAOs exist in two isoforms: MAO A, which mainly decomposes serotonin and dopamine (its role being somewhat region-dependent [5]), and MAO B, which predominantly metabolizes benzylamine and phenylethylamine [6,7]. The human A and B isoforms are quite similar, sharing 70% of their amino acid sequence and the same flavin adenine dinucleotide (FAD) prosthetic group. The MAO X-ray structure [8,9] allows for molecular simulation of the reaction mechanism and rational inhibitor design.

MAO irreversible inhibitors like selegiline, rasagiline, and clorgyline are in clinical use, but the reaction mechanism of irreversible inhibition is not entirely known [10,11]. The mechanism of the reaction catalyzed by MAO enzymes is the subject of heated debates [12,13]. Recent experimental and computational studies provide strong evidence that the rate-limiting step mechanism is hydride transfer from the methylene group vicinal to the amino group of the substrate to the flavin moiety of MAO, as suggested by Vianello et al. [3].

Molecular simulation on the multiscale (QM/MM) level is a powerful tool that provides information about the transition state and the activation free energy. As such, it allows for treatment of enzyme reactions and reactions in solution [14,15,16,17,18,19].

We applied multiscale QM/MM simulations using the empirical valence bond (EVB) method developed by Warshel [14,20,21], in order to obtain insight into MAO A-catalyzed decomposition of benzylamine. The EVB method is based on the quantum coupling of valence states, representing the reactants and the products, with molecular mechanics representing the surroundings. The formalism uses empirical parameters, which must be calibrated (either experimentally or with quantum computational methods) using a reference reaction of the same mechanism in a reference medium (usually water). For the purposes of this article, we calibrated our reaction to the experimental values [22]. The biggest advantage of EVB is that it is computationally inexpensive and allows for well-converged free energy profiles.

In this study, we considered the rate-limiting step of benzylamine decomposition catalyzed by monoamine oxidase A and elucidated the hydrogen/deuterium (*H*/*D*) kinetic isotope effect (KIE). Benzylamine is one of the experimentally preferred MAO substrates, because most of the endogenic substrates, such as dopamine and norepinephrine [23,24,25], are prone to autoxidation. The EVB methodology, in conjunction with the quantum classical path (QCP) method, was applied in order to calculate the free energy barriers for both the hydrogen and the deuterium isotopomer, and a critical comparison with the experimental values was made.

## 2. Computational Details

The calculations were performed using the Q6 program package [26] with the OPLS-AA force field [27,28,29]. The *H*/*D* kinetic isotope effect reflects the quantum character of nuclear motion. A method of choice is to quantize nuclear motion for a few relevant atoms that play an important role in the studied reaction. The rest of the system may be studied by using the classical description of nuclear motion. In our work, path integration was employed for all EVB atoms (benzylamine and the truncated flavin moiety) by using quantum classical path methodology [30].

The basis of the QCP approach is a functional dependence between the nuclear wave-function and the ring of quasiparticles that are propagated on the “quantum mechanical” potential *U*_qm_, expressed as follows:

(1) $$U_{qm}=\overset{p}{\underset{k=1}{\sum }}\frac{1}{2p}M\Omega^{2}\Delta x_{k}^{2}+\frac{1}{p}U\left(x_{k}\right)$$

where $\Delta x_{k}=x_{k+1}-x_{k}$, where $x_{p+1}=x_{1}$. The last equality ensures that the ring of particles is closed. Moreover, Ω=p/ℏβ, while *M* is the mass of the quantum atom and *U* is the actual potential used in the simulation. The total quantum mechanical partition function can be obtained by running classical trajectories of the quasiparticles with the potential *U_qm_* and $\beta =1/k_{B}T$.

It should be pointed out that a big disadvantage of “on-the-fly” path integral simulations is that they are demanding because of the poor equilibration between quantum and classical particles. On the other hand, the QCP approach is based on propagating classical trajectories on a classical potential energy surface of the reacting system. The atom positions are used to generate the centroid positions for the quantum mechanical partition function. This approach is effective and suitable for massive parallelization. The main equation of this approach defines the quantum mechanical partition function, expressed as follows:

(2) $$Z_{qm}\left(\bar{x}\right)=Z_{cl}\left(\bar{x}\right)<<exp{\left\{-\left(\frac{\beta }{p}\right)\sum_{k}U\left(x_{k}\right)-U\left(\bar{x}\right)\right\}>}_{fp}>{}_{U},$$

where $\bar{x}$ is the centroid position, ${<\cdots >}_{fp}$ designates an average over the free particle quantum mechanical distribution obtained with the implicit constraint that $\bar{x}$ coincides with the current position of the corresponding classical particle, and ${<\cdots >}_{U}$ designates an average over the classical potential *U*. The quantum mechanical free energy correction calculated from the partition function reads $A_{QM}=-1/\beta ln\left(Z\right)$.

**Figure 1:**
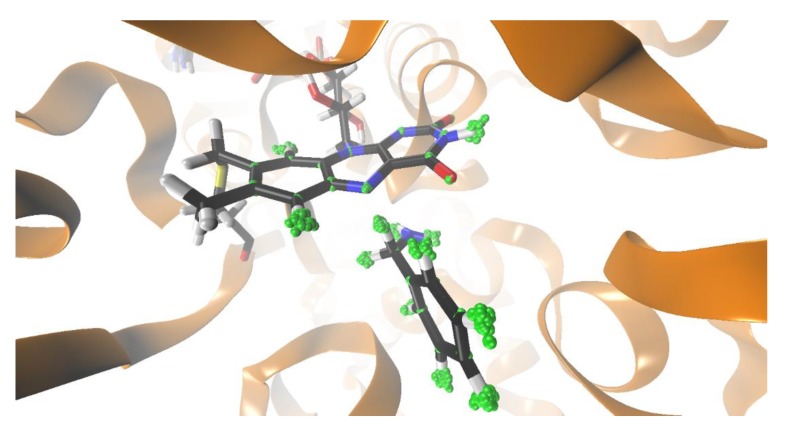
Structure of monoamine oxidase A (MAO A) active site with benzylamine substrate. The active site flavin adenine dinucleotide (FAD), as well as benzylamine, are depicted with sticks. Atom motion for flavin and benzylamine was quantized using the quantum classical path (QCP) approach. The quantized atoms are represented as green beads.

The molecular dynamics simulations were performed under the same conditions as in our previous work, using the standard EVB free energy perturbation/umbrella sampling (FEP/US) procedure [31,32,33]. The EVB region is electronically polarizable, because the atomic charges of the polar enzymatic environment are included in the Hamiltonian, and the atomic charges of the EVB region change. In this respect, the full dimensionality of the enzyme was included. The model of solvated MAO A was built based on the X-ray crystal structure of MAO A in complex with a reversible inhibitor (Protein Data Bank entry 2Z5X [9]), with the harmine molecule removed and the benzylamine molecule manually docked into the active site. The EVB region consisted of the truncated FAD and the substrate benzylamine, as shown in Figure 1. The system was solvated in a spherical water droplet with a radius of 30 Å (centered in the reactive region), subject to surface-constrained, all-atom solvent boundary conditions [34]. In turn, the spherical droplet was placed in a continuum with the dielectric constant of water. A cut-off of 10 Å was used for protein–protein interactions, and the local reaction field was applied for long-range interactions beyond 10 Å. All interactions between the EVB region and the solvated protein were included. First, the system was carefully equilibrated in 10 steps, slowly raising the temperature from 1 K to 300 K, increasing the time step from 0.1 fs to 1 fs, and gradually minimizing the restraints. After a total simulation time of 1.28 ns, a final equilibrated structure was produced, which was the starting point for 20 distinct replicas, which were each additionally equilibrated for 50 ps at 300 K. The system was converted from the state of reactants to the products in 51 mapping frames, each of which consisted of a molecular dynamics simulation of 10 ps, using a 1 fs time step, for a total simulation time of 510 ps (and yielding 5100 snapshots of our system) for each replica. The interaction between the donor α-carbon of the substrate and the acceptor nitrogen of the flavin was distance-restrained by using different equilibrium distance values and force constants. We varied the distance from 2.5 Å to 3.0 Å and the force constant from 1.0 kcal (mol^−1^ × Å^−2^) to 10.0 kcal (mol^−1^ × Å^−2^).

Quantization of nuclear motion for the benzylamine and the flavin moiety was performed using 32 beads for each quantized atom (Figure 1). In this way, the motion of 36 atoms was quantized in the three-dimensional physical space. The quantization was performed *a posteriori* on snapshots extracted from the FEP trajectories (5100 snapshots per replica). For each of the classical coordinate snapshots, the necklace of beads was first equilibrated for 10 steps, and the ring polymer configurations were sampled for 100 steps. Both H and D isotopomers were considered. When performing calculations for the D isotopomer, both hydrogen atoms of the methylene group were replaced by deuterium in order to facilitate comparison with the experiment.

## 3. Results and Discussion

The calculated *H*/*D* kinetic isotope effect values are displayed in Table 1. In order to explore the robustness of the applied simulation protocol, we analyzed the influence of the applied distance restraint on the KIE, which is calculated via the difference in free energy of activation (Δ*G*^#^) for species involving *D* and *H*, expressed as follows:

(3) KH/DIE=eΔG#(D)−ΔG#(H)kBT

The calculated KIE values ranged between 5.9 and 6.5, which is in reasonable agreement with the experimental values measured by Miller and Edmondson, which ranged between 6 and 13, depending on the substituted benzylamine used [22]. For the MAO A-catalyzed oxidation of the unsubstituted benzylamine substrate, the kinetic isotope effect value was experimentally determined to be 11.5. Considering the exponential relationship between the kinetic isotope effect and the free energy barrier, this means that our calculated values are still in good agreement with the experimentally determined one (a difference of approximately 1 kcal/mol on the free energy scale). Kästner and coworkers [35,36] presented extensive mechanistic studies of the MAO B system and examined the possible pathways of the considered reaction with benzylamine. These extensive mechanistic studies indicate that in comparison to that of MAO B, the protein environment of MAO A enhances the polar nucleophilic character of the mechanism. The authors reported the KIE to be between 2.4 and 3.7. The *H*/*D* KIE was calculated by diagonalizing the mass-weighted Hessian for the reactant well and the transition state, respectively, by considering the reactive QM part of the system described with density functional theory (DFT). Zero-point energy corrections were calculated for both isotopomers. The “through the barriers” contribution to tunneling was calculated by using a one-dimensional Eckart model, producing the analytical solution. In contrast, the presently used QCP method is a unifying approach that accounts for energy corrections due to nuclear quantum effects, including tunneling through the barrier.

The calculated isotope effect shows reasonably good agreement with the experimental kinetic measurements [22]. Applying different restraint parameters for the distance between the donor α-carbon atom of the substrate and the acceptor nitrogen atom of the flavin produced similar results, with the KIE values ranging from 5.9 to 6.3, proving the robustness of the applied simulation protocol. The studied enzymatic reaction proceeded about six times faster for the H isotopomer than it did for the D isotopomer, clearly showing the relevance of the quantum nature of nuclear motion for reactivity. We anticipate that a comparable *H*/*D* kinetic isotope effect is present in the reference reaction in aqueous solution. Unfortunately, experimental kinetic data for the reference reaction is not available, mainly because the reaction is too slow to be studied. Even so, it seems that nuclear tunneling does not play a major role in the catalytic function of the MAO A enzyme. This is in accordance with Warshel’s claim that preorganized electrostatics are the only relevant factors responsible for enzyme catalysis, while nuclear quantum effects and dynamical effects do not contribute to catalysis [37]. Since the experimental *H*/*D* KIE effect depends on the oxygen level, it could be interesting to study the flavin regeneration reaction with oxygen where hydrogen peroxide is formed.

## 4. Conclusions

In this study, we calculated the H/D kinetic isotope effect values of 6.45 ± 1.37 for MAO A-catalyzed decomposition of benzylamine by using path integration, which is close to experimental values. The rate-limiting step in this reaction was abstraction of the hydride anion from the substrate methylene group vicinal to the amino group. In addition, the path integral implementation QCP algorithm proved to be a computationally efficient and robust method of choice for the quantization of nuclear motion in enzyme catalysis. Recently, deuterated drugs entered clinical practice and have been gaining significant research attention [38]. We are sure that the presently applied methodology is applicable to studying the stability of deuterated drugs.

## Figures and Tables

**Table 1:** Calculation of hydrogen/deuterium (H/D) kinetic isotope effect for MAO A-catalyzed decomposition of benzylamine. Activation free energies for species with hydrogen (H) and deuterium (D) in kcal/mol are given. The reported results are averages over 20 replicas with the exception of the simulation of 2550 ps, where only 10 replicas were applied.

FEP Lengths	Applied Restraint		ΔG^#^(H)	ΔG^#^(D)	H/D KIE
[ps] Per Replica	Distance [Å]	Force Constant [(kcal (mol^−1^ × Å^−2^)]	[kcal/mol]	[kcal/mol]	
510	0–2.5	1	17.17	18.26	6.34 ± 1.55
510	0–2.5	5	17.23	18.32	6.34 ± 1.42
510	0–3.0	1	17.23	18.29	6.03 ± 1.44
510	0–3.0	5	17.22	18.29	6.03 ± 1.55
510	0–3.0	10	17.18	18.28	6.45 ± 1.37
2550	0–3.0	5	17.27	18.27	5.93 ± 1.68
